# Novel biomarker combination improves the diagnosis of serious bacterial infections in Malawian children

**DOI:** 10.1186/1755-8794-5-13

**Published:** 2012-05-04

**Authors:** Adam D Irwin, Fiona Marriage, Limangeni A Mankhambo, IPD Study Group, Graham Jeffers, Ruwanthi Kolamunnage-Dona, Malcolm Guiver, Brigitte Denis, Elizabeth M Molyneux, Malcolm E Molyneux, Philip J Day, Enitan D Carrol

**Affiliations:** 1 Department of Women’s and Children’s Health, University of Liverpool, Liverpool, UK; 2 Malawi-Liverpool-Wellcome Trust Clinical Research Programme, Blantyre, Malawi; 3 Centre for Integrated Medical Genomic Research, University of Manchester, Manchester, UK; 4 Department of Biostatistics, University of Liverpool, Liverpool, UK; 5 Health Protection Agency North West, Manchester Medical Microbiology Partnership, Manchester, UK; 6 Department of Paediatrics, University of Malawi College of Medicine, Blantyre, Malawi; 7 Institute of Child Health, University of Liverpool, Alder Hey Children’s NHS Foundation Trust, Eaton Road, Liverpool, L12 2AP, UK

**Keywords:** Biomarker, Bacterial infection, Transcriptomics, Diagnosis, Protein, Biology, Procalcitonin, Resistin, Neutrophil-associated gelatinase

## Abstract

**Background:**

High throughput technologies offer insight into disease processes and heightens opportunities for improved diagnostics. Using transcriptomic analyses, we aimed to discover and to evaluate the clinical validity of a combination of reliable and functionally important biomarkers of serious bacterial infection (SBI).

**Methods:**

We identified three previously reported biomarkers of infection (neutrophil gelatinase-associated lipocalin (NGAL), granulysin and resistin) and measured gene expression using quantitative real-time PCR. Protein products related to the three transcripts were measured by immunoassays.

**Results:**

Relative gene expression values of NGAL and resistin were significantly increased, and expression of granulysin significantly decreased in cases compared to controls. Plasma concentrations of NGAL and resistin were significantly increased in children with confirmed SBI compared to children with no detectable bacterial infection (NBI), and to controls (287 versus 128 versus 62 ng/ml and 195 versus 90 versus 18 ng/ml, respectively, p < 0.05). Plasma protein concentrations of NGAL and resistin were significantly increased in non-survivors compared to survivors (306 versus 211 and 214 versus 150 ng/ml, p = 0.02). The respective areas under the curve (AUC) for NGAL, resistin and procalcitonin in predicting SBI were 0.79, 0.80 and 0.86, whilst a combination of NGAL, resistin and procalcitonin achieved an AUC of 0.90.

**Conclusions:**

We have demonstrated a unique combination of diagnostic biomarkers of SBI using transcriptomics, and demonstrated translational concordance with the corresponding protein. The addition of NGAL and resistin protein measurement to procalcitonin significantly improved the diagnosis of SBI.

## Background

Serious bacterial infection (SBI) remains an important contributor to mortality and morbidity in children, particularly in resource-poor countries. Child deaths attributable to invasive *Streptococcus pneumoniae* and *Haemophilus influenzae* type b infections are estimated to equal those collectively attributable to HIV, tuberculosis and malaria [1,2]. The prompt recognition of SBI in children is challenging clinically, especially so in the context of co-infection with malaria and HIV [3], and is of the utmost importance in ensuring both effective, and rational antibiotic treatment. Diagnostic uncertainty in bacterial sepsis is compensated by the overuse of antibiotics in self-limiting infections, leading to antibiotic resistance.

Biomarkers may be used alone or in combination to allow classification of an individual to a unique group with defined characteristics. Biomarkers which reflect the host response to infection are useful adjuncts to good clinical acumen and improved molecular diagnostic techniques in confirming SBI. Advances in transcriptomics and proteomics have provided basis for biomarker panels unravelled from these technologies, that might be useful in differentiating sepsis-associated inflammation from non-infectious inflammation [4]. An improved understanding of the mechanism of sepsis, and the ability to identify and quantify key mediators involved in its pathophysiology, offers the prospect of reliable diagnostic and prognostic tools. Transcription profiling has been used in other diseases to identify candidate biomarkers [5-7].

Over 170 biomarkers have been related to sepsis, but relatively few have been evaluated as diagnostic markers. A comprehensive review on sepsis biomarkers, concluded that it is unlikely that a single ideal biomarker will ever be found, and that a combination of biomarkers may be more effective [8]. Many of the markers so far investigated are costly and time-consuming to measure. Studies of panels of biomarkers have yielded encouraging results, but none of them have potential to be measured as an affordable point-of- care (POC) test.

The performance of biomarkers is traditionally assessed using receiver operator characteristic (ROC) curves. ROC curves evaluate the discrimination of a test (or combination of tests), but may be insensitive to the addition of a new biomarker, especially if a good biomarker is already included in the model. Net reclassification improvement (NRI) quantifies improvement in the model as a result of adding one or more new biomarkers. This approach has been used in cardiovascular disease [9] and acute lung injury [10] to improve the accuracy of risk prediction.

Resistin is an adipokine first related to insulin resistance in mice [11]. In humans, resistin expression occurs predominantly in macrophages, monocytes and neutrophils [12,13]. Circulating resistin levels peak 8-16 h after the administration of LPS in healthy subjects, mediated by pro-inflammatory cytokines and NF-κB [14]. Resistin is significantly elevated in intensive care patients with sepsis [15], and levels correlate with severity of disease [16,17].

Neutrophil gelatinase-associated lipocalin (NGAL) – or lipocalin 2 –has a role in innate immunity through its ability to bind siderophores, required by many bacterial pathogens to scavenge iron from the host [18]. Like resistin, it is highly upregulated by inflammatory stimuli via NF-κB [19]. NGAL is more significantly elevated in sepsis than in the systemic inflammatory response syndrome (SIRS) [20-22]. In a large study of adult patients with suspected sepsis presenting to an emergency department, the optimal 3-marker panel was NGAL, protein C, and interleukin-1 receptor antagonist. The area under the curve for the accuracy of the sepsis score derived from these three biomarkers was 0.80 for severe sepsis, 0.77 for septic shock, and 0.79 for death [23].

Granulysin is an antimicrobial protein co-localised with perforin in the granules of cytotoxic T lymphocytes (CTLs) and natural killer (NK) cells. It has a direct cytotoxic effect on numerous extracellular pathogens, and is postulated to kill intracellular organisms such as *Mycobacterium tuberculosis* in conjunction with perforin [24]. Granulysin levels correlate inversely with disease activity in *M. tuberculosis* and normalise with treatment [25]. Granulysin is transiently elevated in acute viral infections, and has been suggested as a marker of host cellular immunity [26].

We aimed to identify differentially regulated biomarker transcripts in children with SBI using microarray technology. Three previously identified biomarkers were validated by quantitative real-time PCR in an independent prospective cohort. In order to determine the diagnostic utility of the biomarkers to predict SBI, we measured the proteins of the differentially regulated biomarker genes in plasma samples, to determine if they could reliably predict SBI, or outcome from SBI.

## Results and discussion

### Results

**Figure 1:**
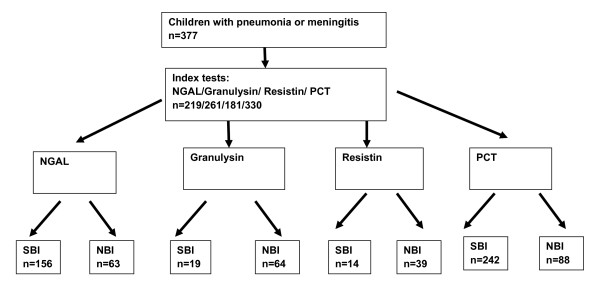
**Flow diagram showing the number of patients undergoing index tests and the number of patients with SBI and NBI.** The numbers of patients that had each measurement of are shown, according to STARD guidelines [27]. All available samples were tested for all four biomarkers in the following order, procalcitonin, granulysin, NGAL, resistin, as long as there was remaining sample. A total of 181 samples were tested across all four assays.

Of the 377 children included in the study, 282 (74.8%) presented with meningitis and 95 (25.2%) presented with pneumonia. The baseline characteristics are summarised in Table 1. There were no significant differences in clinical characteristics (duration of symptoms, previous antibiotics, nutritional status) between children with serious bacterial infection (SBI) and children with signs of severe sepsis but no detectable bacterial infection (NBI), but as expected, the proportion of children presenting with signs of meningitis was significantly higher in the SBI group. The numbers of patients in whom we measured NGAL, resistin, granulysin and procalcitonin are shown in Figure 1, according to STARD guidelines [27]. 

**Table 1:** Characteristics of children with confirmed serious bacterial infection (SBI), those with no detectable bacterial infection (NBI) and controls

	**SBI (n = 280)**	**NBI (n = 97)**	**Controls (n = 15)**	**p value**
**Age (years),*median(IQR)***	2.0 (0.6 – 6.9)	2.5 (1.0 – 5.7)	10.0 (6.0-13.0)	<0.0005
**Males**	154 (55%)	61 (63%)	13/21 (62%)	<0.0005
**Duration of symptoms (days),*median(IQR)***	3 (2 – 5)	3 (2 – 5)	NR	0.76
**Previous antibiotics (%)**	123 (44%)	37 (38%)	NR	0.32
**Meningitis (%)**	235 (84%)	47 (49%)	NR	<0.0005
**Mortality (%)**	68 (24%)	15 (15%)	NR	0.02
**HIV-infected (%)**	145 (52%)	45 (47%)	0 (0%)	<0.0005
**Height for age Z score < − 3 (%)**	42/272 (15%)	16/91 (18%)	ND	0.63
**Weight for height Z score < − 3 (%)**	37/218 (17%)	11/82 (13%)	ND	0.45
**White cell count (WCC) x10**^**9**^**/l*median(IQR)***	12.6 (7.8 – 20.0)	13.8 (10.0 – 20.4)	ND	0.09
**Neutrophil count x10**^**9**^**/l*median(IQR)***	10.6 (4.6 – 16.3)	8.9 (5.5 – 15.5)	ND	0.78

Numeric values are median and interquartile range (IQR). (ND = not done, NR = not relevant in a healthy control). Statistical comparison is between three groups, SBI, NBI and controls, using the Kruskal = Wallis test.

**Table 2:** Performance characteristics of NGAL and resistin as biomarkers of SBI, singly, and in combination with procalcitonin

**Marker**	**Threshold**	**Sensitivity** **(95% CI)**	**Specificity** **(95% CI)**	**PPV** **(95% CI)**	**NPV (95% CI)**	**LR+**	**LR-**
NGAL	100	86.7 (83.2-90.2)	50.0 (44.8-55.1)	78.7 (74.4-82.9)	63.9 (58.9-68.9)	1.735	0.265
Resistin	80	91.4 (88.4-94.3)	51.9 (46.6-57.0)	84.1 (80.4-87.9)	68.3 (63.5-73.1)	1.898	0.166
**In combination with Procalcitonin:**							
PCTNGALResistin	2 100 80	82.7 (76.6-88.8)	76.7 (69.9-83.5)	89.6 (84.6-94.5)	64.7 (57.0-72.4)	3.556	0.226
PCTNGALResistin	1 80 80	86.6 (78.8-90.4)	72.1 (64.8-79.3)	88.0 (82.7-93.2)	66.0 (58.3-73.6)	3.032	0.213

PPV = positive predictive value, NPV = negative predictive value, LR + = likelihood ratio for a positive result, LR- = likelihood ratio for a negative result.

### Relative gene expression

In the biomarker discovery set using microarray, we studied 12 children with serious bacterial infection (HIV-infected non-survivor n = 3, HIV-infected survivor n = 3, HIV-uninfected non-survivor n = 3, HIV-uninfected survivor n = 3), and the control group (n = 3). We identified three genes previously reported to be biomarkers of bacterial infection: resistin (RETN), neutrophil gelatinase associated lipocalin (LCN2) and granulysin (GNLY). The following fold changes were observed in controls compared to cases: Resistin −5.88 (p = 0.06), NGAL −4.19, (p = 0.002) and granulysin 3.14 (p = 0.002). These three genes were chosen because a) there was *a priori* evidence in the literature that they could be markers of infection, b) they had the highest fold changes (>3, <–3), and c) they were all available as commercial ELISAs for measurement of protein in plasma samples.

**Figure 2:**
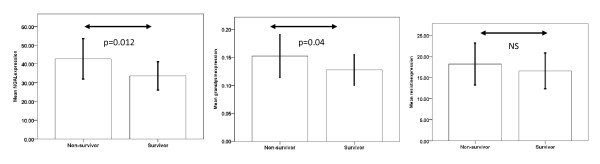
**Bar chart showing relative NGAL, granulysin and resistin expression in survivors and non-survivors.** A total of 176 samples were analysed using RT PCR. The ++ Ct method was used to calculate normalised data [28]. There was over-expression of NGAL and resistin and under-expression of granulysin in cases compared to controls (data not shown). Relative gene expression of NGAL granulysin and resistin were increased in non-survivors compared to survivors Error bars = mean+/−2SE NS = not significant.

In the independent validation set we studied 176 children with serious bacterial infection. Children from the microarray discovery set were excluded. Relative gene expression of resistin and NGAL were significantly increased in cases compared to controls (p < 0.001), and relative gene expression of granulysin was decreased in cases compared to controls. Relative gene expression of NGAL (median 41.2 v 17.7, p = 0.012) granulysin (0.1 v 0.07, p = 0.04) and resistin (13.7 v 11.0, p = 0.26), was increased in non-survivors compared to survivors (Figure 2).

### Plasma concentrations of NGAL, granulysin and resistin in NBI, SBI and controls

**Figure 3:**
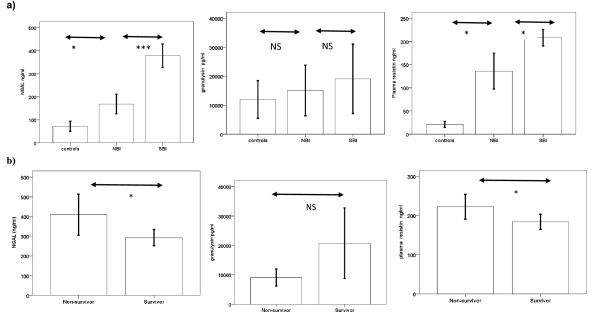
**Bar chart showing NGAL, granulysin and resistin concentrations in a) controls, NBI and SBI and b) in survivors and non-survivors.** A total of 181 samples were tested across all 4 assays. Plasma concentrations of NGAL and resistin were significantly increased in children with SBI compared to NBI, compared to controls. Plasma concentrations of NGAL and resistin were significantly increased in non-survivors compared to survivors. Error bars = mean+/−2SE NS = not significant, *** = p < 0.0005, * = p < 0.05.

After adjusting for HIV status, plasma concentrations of NGAL and resistin were significantly increased in children with SBI compared to NBI, compared to controls (287 v 128 v 62 ng/ml and 195 v 90 v 18 ng/ml, p < 0.05). Plasma concentrations of granulysin were not significantly different between the groups (Figure 3).

### Plasma concentrations of NGAL, granulysin and resistin in survivors and non-survivors

After adjusting for HIV status, plasma concentrations of NGAL and resistin were significantly increased in non-survivors compared to survivors (306 v 211 ng/ml and 214 v 150 ng/ml, p = 0.02). Plasma concentrations of granulysin were not significantly different between the groups (Figure 3).

### Performance of NGAL, granulysin and resistin in predicting SBI

**Figure 4:**
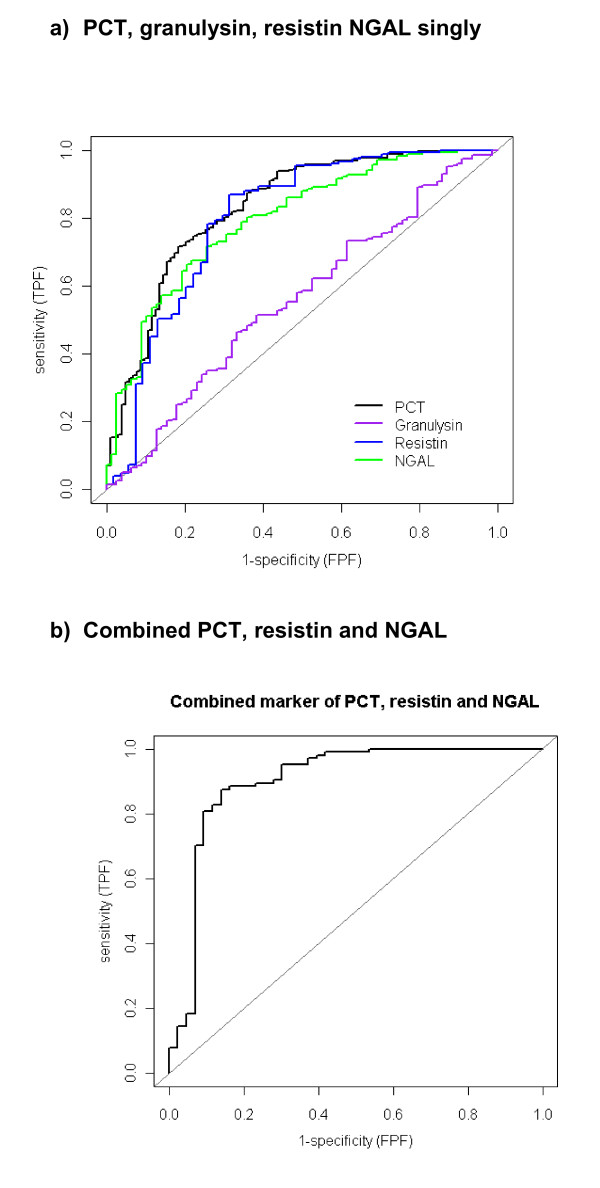
**Receiver operator characteristic curves (ROC) of markers of SBI. a**) procalcitonin (PCT), granulysin, resistin and NGAL as markers of SBI and **b**) combination of biomarkers (procalcitonin (PCT), resistin and NGAL) as markers of SBI.

The performance characteristics (sensitivity, specificity, positive and negative predictive values, and positive and negative likelihood ratios) of NGAL and resistin as biomarkers of SBI, singly, and in combination with procalcitonin are shown in Table 2. The areas under the curve (AUCs) for NGAL, granulysin and resistin in predicting SBI were NGAL 0.79 (95% CI 0.73-0.85), granulysin 0.56 (95% CI 0.48-0.63) and resistin 0.80 (95% CI 0.72-0.88). Procalcitonin, a previously evaluated biomarker in this cohort [29], had an AUC of 0.86 (0.79-0.92). The ROC curves of the four biomarkers and the combination of procalcitonin, resistin and NGAL are shown in Figure 4. 

**Table 3:** Reclassification improvement in new prediction models versus current PCT alone model

**New model**	**Summary NRI (95% CI), p-value**	**NRI for events (95% CI), p-value**	**NRI for non-events (95% CI), p-value**
**PCT + Resistin**	0.50 (0.17, 0.83), 0.0032	−0.04 (−0.22, 0.12), 0.6015	0.54 (0.26, 0.82), 0.0002
**PCT + NGAL**	0.58 (0.29, 0.86), 0.0001	0.01 (−0.15, 0.18), 0.8690	0.56 (0.32, 0.79), <0.0001
**PCT + Resistin + NGAL**	0.81 (0.45, 1.16), <0.0001	0.14 (−0.06, 0.33), 0.1700	0.67 (0.38, 0.97), <0.0001

Event = SBI, non-event = not SBI.

### Additive value of the biomarkers using net reclassification index

The addition of the biomarkers resistin and NGAL to the currently used biomarker, procalcitonin alone, significantly improved prediction of SBI. Granulysin was dropped from the model because of its poor performance as a single biomarker.

The NRI for the new prediction models are shown in Table 3. Including resistin or NGAL or both resistin and NGAL in the current SBI prediction model (i.e.PCT alone) [29] significantly improved the overall performance. In addition, the incorporation of resistin in the current prediction model results in a 4% loss of sensitivity (non-significant), but a (significant) 54% improvement in specificity. The inclusion of NGAL in the current model results in a 1% gain in sensitivity and a (significant) 56% gain in specificity. The inclusion of both resistin and NGAL in the current model results in a 14% gain in sensitivity and a (significant) 67% gain in specificity. 

**Table 4:** Primer sequences and probe numbers for each assay

**Gene**	**Forward Primer Sequence**	**Reverse Primer Sequence**	**Universal Probe Library**
**GNLY**	AGGGTGACCTGTTGACCAAA	CAGCATTGGAAACACTTCTCTG	17
**LCN2**	TCACCTCCGTCCTGTTTAGG	AGGTAACTCGTTAATCCAGGGTAA	61
**RETN**	TGCAGGATGAAAGCTCTCTG	CATGGAGCACAGGGTCTTG	45
**ACTB**	ATTGGCAATGAGCGGTTC	GGATGCCACAGGACTCCAT	11
**B2M**	TTCTGGCCTGGAGGCTATC	TCAGGAAATTTGACTTTCCATTC	42
**GAPDH**	AGCCACATCGCTCAGACAC	GCCCAATACGACCAAATCC	60
**GNB2L1**	GCTACTACCCCGCAGTTCC	CAGTTTCCACATGATGATGGTC	55
**HMBS**	AGCTATGAAGGATGGGCAAC	TTGTATGCTATCTGAGCCGTCTA	25
**HPRT1**	TGACCTTGATTTATTTTGCATACC	CGAGCAAGACGTTCAGTCCT	73
**PGK-1**	CTGTGGCTTCTGGCATACCT	CTTGCTGCTTTCAGGACCA	42
**RPL13A**	GAGGCCCCTACCACTTCC	TGTGGGGCAGCATACCTC	28
**RPL32**	GAAGTTCCTGGTCCACAACG	GCGATCTCGGCACAGTAAG	17
**SDHA**	AGAAGCCCTTTGAGGAGCA	CGATTACGGGTCTATATTCCAGA	69
**TBP**	GCTGGCCCATAGTGATCTTT	CTTCACACGCCAAGAAACAGT	3
**YWHAZ**	CGTTACTTGGCTGAGGTTGC	TGCTTGTTGTGACTGATCGAC	9

12 endogenous transcripts (ACTB, B2M, GAPDH, GNB2L1, HMBS, HPRT1, PGK-1, RPL13A, RPL32, SDHA, TBP and YWHAZ) were screened and analysed using the GeNORM algorithm [28].

## Discussion

The diagnosis of SBI is complicated by its variable and non-specific clinical signs and symptoms, and a failure to recognise and promptly treat SBI results in significant morbidity and mortality. The search for accurate biomarkers to influence clinical practice has become paramount for the treatment of sepsis, but to date the search has been disappointing [8]. We have demonstrated concordance between transcript and protein expression for two out of three biomarkers of SBI differentially regulated in microarray screening, validation using quantitative RT-PCR, and measurement of protein concentrations in plasma samples. The discrepancy between RNA expression and protein concentration for granulysin can be explained by a) post-translational modification, b) reduced expression of granulysin in HIV-infected individuals [30] (although after adjusting for HIV the results are the same) and c) delay in granulysin production in HIV-infected individuals due to defective signalling in HIV [31].

Our combination of three biomarkers (resistin, NGAL and procalcitonin) demonstrates good discrimination between SBI and NBI. Adding resistin and NGAL to procalcitonin demonstrates a net reclassification improvement of 81%. To our knowledge, this is the first study to identify a combination of biomarkers for the detection of SBI using a systems biology approach, and to validate diagnostic accuracy. Our data suggest possible inter-relationships between pathways involving resistin, cytotoxic T cell activity and lipocalin-2, and a significant role for these proteins in the host defence against bacterial infection. The exact mechanisms require further detailed study. Resistin has been shown to attenuate both cytokine production and T cell activity in a dendritic cells stimulated with lipoteichoic acid [32], and adiponectin (another adipokine) is a negative T cell regulator [33].

Transcription profiling has been used in other infectious diseases to identify biosignatures of disease groups [5-7]. The use of multiple biomarkers allows the capture of multi-dimensional patterns and pathways, such as those that can occur in complex biological processes like sepsis, and is therefore more robust [34]. Our study is now undergoing further validation in a prospective cohort of febrile children presenting to the emergency department of a large tertiary hospital in England.

In this study, resistin and NGAL provided good discrimination of SBI from NBI (AUC: 0.80 and 0.79 respectively). Individually, neither performed as well as procalcitonin in our previous study [29]. A combination of the three biomarkers performed better (AUC: 0.90) than an optimal 3-marker panel (NGAL, protein C, and interleukin-1 receptor antagonist) measured in North American adults presenting to an emergency department with suspected sepsis [23]. As measured by the AUC, our combination of biomarkers resulted in a 10% improvement in clinical diagnostic accuracy. A more helpful measure however is the NRI. Clinical risk reclassification describes how a new marker or combination of markers may add to predictive models for clinical use, and the NRI can be used to more formally assess clinical utility. An NRI of 81% is a highly significant improvement in reclassification for clinical management.

Our study exemplifies translational medicine. We have used transcriptomics to develop a better understanding of biomarkers related to the disease process, and to identify potent biosignatures for accurate clinical decision-making. Obstacles to the pipeline for diagnostics have been reported at the “front end” i.e. early stages of discovery and development, and also later, with lack of samples for validating and testing biomarkers [35]. Our study has overcome both these barriers, and is a major step in addressing the pipeline problem in the diagnosis of SBI. If our findings are confirmed prospectively, and if the unique combination of three protein biomarkers could be combined in POC device at low cost, then there is great potential for such a POC device. In primary and secondary care, and in resource-poor settings, POC testing could reduce delays in diagnosis thereby reducing morbidity and mortality, and minimise unnecessary hospital admission and antibiotic prescribing.

## Conclusions

There are currently no rapid diagnostic tests measuring panels of biomarkers of SBI. Our study demonstrates for the first time that a small panel derived from disease-related biological processes, is likely to be successful. The addition of resistin and NGAL protein measurement to procalcitonin significantly improves classification. We have identified a unique combination of biomarkers of SBI which could help guide antibiotic management and decisions on referral to hospital. Our data demonstrate exciting improvements in classification, which need to be validated prospectively in a larger cohort.

## Materials and methods

### Study setting and population

Between April 2004 and October 2006, we prospectively enrolled children who presented to the Accident and Emergency Department and the Admissions Unit of Queen Elizabeth Central Hospital, Blantyre, Southern Malawi. The study setting, population and characteristics of study patients have been described previously [29].

The primary outcome measure was bacteriological confirmation of infection (SBI) and the secondary outcome measure was death/survival in hospital. Afebrile children, without malaria parasitaemia, from the same villages as the cases, were used as controls. All controls were HIV-uninfected. Ethical approval for this study was granted from The College of Medicine Research Committee (COMREC), Malawi and The Liverpool School of Tropical Medicine Research Ethics Committee. Parents or guardians gave written informed consent for children to enter the study.

### Definitions

#### *Cases (n = 377)*

Children who presented with signs and symptoms of meningitis or pneumonia, as defined previously [29].

#### *Healthy controls* (n = 15)

Healthy afebrile children from the same villages as the cases, who had no malarial parasites on blood film. Controls were selected by parents or guardians in the neighbourhood of the index case as part of a larger study investigating genetic susceptibility in IPD [36]. In a small number of children, parental consent was also given to take venous samples for biomarker determination.

#### *Serious bacterial infection (SBI)* (n = 280)

Children who presented with either bacterial meningitis or bacterial pneumonia, in whom a bacterial pathogen was identified by culture, polysaccharide antigen test or PCR (*Streptococcus pneumoniae, Neisseria meningitidis, and Haemophilus influenzae b*).

#### *No detectable bacterial infection (NBI)* (n = 97)

Children who presented with bacterial meningitis or bacterial pneumonia, but who were negative for any bacteria on culture, polysaccharide antigen test or PCR (*S. pneumoniae, N. meningitidis, and H. influenzae b*).

### Microbiological methods and malaria diagnosis

Blood, CSF and lung aspirate culture and PCR were performed using standard microbiological techniques as previously described [37,38] . Blood films were examined by microscopy according to best standard practice of district-hospital laboratories in Africa.

### RNA extraction and quantification

Total RNA was extracted from whole blood using an optimised method for the PAXgene^TM^ blood RNA kit (Qiagen, West Sussex, UK), as previously described [28]. The RNA was concentrated by precipitating overnight at −20°C with 2 μl linear acrylamide (5 mg/ml), (Ambion, Warrington, UK), 0.5 volumes 7.5 M Ammonium acetate (Sigma, Dorset, UK) and 2.5 volumes ice cold 100% ethanol. The samples were centrifuged at 13,000 g, 4°C for 30 minutes. The RNA pellet was washed twice with 0.5 ml ice cold 80% ethanol, each time centrifuging at 4°C for 10 minutes at 13,000 g. The pellet was air dried for approximately 5 minutes and re-suspended in 11 μl of nuclease free water.

The total RNA concentration (ng/μl) and ratios (260/280 and 260/230) were measured using a NanoDrop ND-100 UV–vis spectrophotometer (Labtech International, Ringmer, UK) and the RNA integrity was assessed using the Agilent 2100 Bioanalyser (Agilent Technologies, Edinburgh, UK) pre- and post-concentration.

### Reverse transcription for qPCR

RNA samples were DNAse treated (DNA Free, Ambion, Warrington, UK] to remove any contaminating genomic DNA. RNA (1 μg) was reverse transcribed with SuperScript II^TM^ RNase H^-^ Reverse Transcriptase and oligo (dT)_12–18_ (500 μg/ml) following the manufacturer’s guidelines ((Invitrogen, Paisley,UK). The cDNA was stored at −40°C until required.

### Real-time quantitative PCR measurement of target genes

The Human Universal Probe Library system (Roche, Burgess Hill,UK) employing proprietary locked nucleic acids (LNA) analogues was used for real-time quantitative PCR (qPCR) to measure expression levels in genes of interest. Using the Roche Online Assay Design Centre, specific primers and an associated probe were selected for the reference and target transcripts. Gene expression was determined using real time qPCR on a Roche LightCycler 480. Assays were designed using the Roche Universal ProbeLibrary system. Table 4 shows the primer sequences and the probe number for each assay. Assays were run in triplicate 10 μl reactions in 384 well plates. Each well contained 5 μl LightCycler 480 Probes Master (2x concentration), 0.1 μl each primer (20 μM), 0.1 μl probe (10 μM), 0.7 μl water and 4 μl of cDNA (1:40 dilution). The thermocycling conditions were: 95°C for 5 minutes, then 50 cycles of 95°C for 10 seconds and 60°C for 30 seconds, a final cooling step of 40°C for 10 seconds completes the program. To compensate for variations in cell number, RNA isolation, reverse transcription and PCR amplification efficiency, 12 endogenous transcripts (ACTB, B2M, GAPDH, GNB2L1, HMBS, HPRT1, PGK-1, RPL13A, RPL32, SDHA, TBP and YWHAZ) were screened and analysed using the GeNORM algorithm [39], HPRT1, GAPDH and YWHAZ were selected as suitable reference genes. A total of 176 randomly selected samples were analysed using qPCR. The amounts of target genes expressed in a sample are normalized to the average of the three endogenous controls. This is given by ΔC_T_, where ΔC_T_ is determined by subtracting the average endogenous gene C_T_ value from the average target gene C_T_ value. [C_T_ target gene – C_T_ average (endogenous gene)]. The calculation of ΔΔC_T_ involves subtraction of ΔC_T_ value for the controls from the ΔC_T_ value for the cases [ΔC_T_ target gene_(case)_ – ΔC_T_ target gene_(control)_. 2^-ΔΔCt^ is the relative expression of the target gene in cases compared to controls [28].

### NGAL, resistin and granulysin measurement by ELISA

NGAL (R&D Systems, Abingdon, UK), resistin (Universal Biologicals, Cambridge, UK) and granulysin (ABO Swiss, Xiamen, China) were measured in plasma samples using commercially available sandwich enzyme immunoassays according to the manufacturers’ instructions. Assays were performed on plasma samples if there was enough sample for the different assays.

### Statistical analysis

Basic analysis was performed using SPSS for Windows, version 15.0, (Illinois, USA), and ROCR and Hmisc packages in R ( http://www.r-project.org/) were used in summarising the performances of biomarkers and computing the net reclassification improvement (NRI). The data, when plotted, did not follow a normal distribution, and therefore the Mann Whitney Test was used to compare distributions. Receiver operator characteristic (ROC) curves were used to determine the areas under the curve (AUCs) with 95% confidence intervals for the biomarkers to predict SBI and death. The laboratory assays for bacteriological confirmation and the markers of infection were performed by investigators blinded to the clinical data. The clinicians involved in managing the cases were not involved in performing any of the laboratory assays.

Net reclassification improvement (NRI) [40] was used to measure the additive value of measuring multiple biomarkers and to quantify improvement in performance over procalcitonin alone, which has previously been shown to be the best biomarker of SBI in Malawian children with signs of severe sepsis [29]. The magnitude of NRI is more important than the statistical significance, therefore we reported NRI with confidence intervals, as well as significance values [40].

The study was reported according to STARD guidelines which includes method of recruitment of patients, orders of test execution, and numbers of patients undergoing the tests under evaluation and the numbers of patients with the reference standard [27] (Figure 1).

## Competing interests

All authors state that they do not have any conflicts of interest to declare.

## Authors' contributions

FM conceived and designed experiments, performed laboratory analysis, and helped draft the manuscript. ADI performed statistical analysis, and drafted the manuscript. LAM recruited patients and collected data. IPD Study Group recruited patients and collected data. GJ performed laboratory analysis. RK-D performed statistical analysis and contributed to writing the manuscript. MG performed microbiology analysis and helped draft the manuscript. BD performed microbiology analysis. EMM contributed to writing the manuscript. MEM contributed to writing the manuscript. PJD conceived and designed experiments contributed to writing the manuscript. EDC conceived and designed experiments, performed statistical analysis, and drafted the manuscript. All authors have read and approved the final manuscript.

## Pre-publication history

The pre-publication history for this paper can be accessed here:

http://www.biomedcentral.com/1755-8794/5/13/prepub
